# Nutritional Ketosis Increases NAD^+^/NADH Ratio in Healthy Human Brain: An *in Vivo* Study by ^31^P-MRS

**DOI:** 10.3389/fnut.2018.00062

**Published:** 2018-07-12

**Authors:** Lijing Xin, Özlem Ipek, Maurice Beaumont, Maya Shevlyakova, Nicolas Christinat, Mojgan Masoodi, Norman Greenberg, Rolf Gruetter, Bernard Cuenoud

**Affiliations:** ^1^Center for Biomedical Imaging, Ecole Polytechnique Fédérale de Lausanne, Lausanne, Switzerland; ^2^Clinical Development Unit, Nestlé Research Center, Lausanne, Switzerland; ^3^Nestlé Institute of Health Sciences SA, Lausanne, Switzerland; ^4^Nestlé Health Science, Epalinges, Switzerland

**Keywords:** ketones, NAD, brain energy metabolism, medium chain triglycerides, hydroxybutyrates, phosphorus magnetic resonance spectorscopy, nutritional sciences

## Abstract

Ketones represent an important alternative fuel for the brain under glucose hypo-metabolic conditions induced by neurological diseases or aging, however their metabolic consequences in healthy brain remain unclear. Here we report that ketones can increase the redox NAD^+^/NADH ratio in the resting brain of healthy young adults. As NAD is an important energetic and signaling metabolic modulator, these results provide mechanistic clues on how nutritional ketosis might contribute to the preservation of brain health.

## Introduction

Brain relies on blood glucose as its predominant energy source. Interestingly, while brain glucose utilization decreases in mild cognitively impaired elderly and in Alzheimer's disease patients, ketone metabolism remains intact (1). Interventions using ketones or their precursors have shown therapeutic potential in several neurometabolic disorders (2), demonstrating the possible value of ketones as an alternative source of brain energy. The two main ketones, beta-hydroxybutyrate (BHB) and aceto-acetate (AcA), are actively transported to the brain by the monocarboxylic transporter 1 (MCT1), resulting in brain levels directly proportional to their blood concentrations (3). They are then further metabolized to Acetyl-CoA and enter the Krebs cycle to generate ATP (2). In Alzheimer's disease patients, it has been recently shown that an increase in blood ketones can restore part of the brain glucose energy deficit (4). Remarkably, under healthy homeostatic conditions, an increase in brain ketones proportionally decreases brain glucose utilization (5).

It is not clear if a shift in energy substrate from glucose to ketones has any further benefit or metabolic consequence in healthy human brain beyond providing energy. For example, the energy production through ketones metabolism toward acetyl-CoA generation is distinct from using glucose and requires a lower utilization of the oxidized form of nicotinamide adenine dinucleotide (NAD^+^). To produce two Acetyl-CoA molecules, one molecule of glucose requires the conversion of 4 NAD^+^ molecules to NADH, the reduced form of NAD (6). Synthesis of Acetyl-CoA from AcA requires no NAD^+^, while the conversion of BHB to AcA requires one NAD^+^. Therefore, one can speculate that an increase in ketolysis may spare NAD^+^ and lead to a greater brain NAD^+^/NADH ratio, a potential mechanism that is becoming recognized (7, 8). When healthy rats were fed with a ketogenic diet, regional increase of brain NAD^+^/NADH was observed already after 2 days and sustained for 3 weeks (8). In a mice model of ischemic stroke, injection of ketones after ischemia induced by transient middle cerebral artery occlusion improved neurological and mitochondria functions, and increased brain NAD^+^/NADH ratio (9). As NAD^+^ is also a critical co-substrate for enzymes that play key roles in many biological processes such as energy metabolism, antioxidation, gene expression, aging, calcium homeostasis, and cell death (10), preserving NAD^+^ concentration may contribute to maintaining health.

Therefore, to test if ketones can increase the brain NAD^+^/NADH ratio in human or affect other energy metabolism pathways, we conducted a phosphorous magnetic resonance spectroscopy (^31^P-MRS) study at 7 tesla, where brain energy related metabolites, redox state, and enzymatic activities were assessed before and after a nutritional ketogenic intervention. Recent advances in quantification of human brain NAD by ^31^P MRS methods allow for direct quantification of NAD^+^ and NADH concentrations despite their relatively low level, making it a unique and non-invasive methodology (11–13). To raise blood ketones, oral intake of Peptamen®, a complete liquid nutrition product containing high amount of medium chain triglycerides (MCT), was used. MCT are efficient ketone precursors when administered by oral bolus (2, 3, 14). They are rapidly digested, and the resultant free medium chain fatty acids (MCFAs) bypass the normal long-chain fatty acid digestion and absorption processes and are absorbed directly into the portal vein to reach the liver where they are extensively metabolized leading to a significant increase in blood ketones level.

## Materials and methods

### Investigational product

250 mL Peptamen®1.5 Vanilla (Nestlé Health Science SA) was used for both pharmacokinetic and ^31^P MRS studies. This is a complete liquid nutrition product containing 10 g MCT, with a caloric density of 1.5 kcal/mL [macronutrient amount in g/250 mL and caloric distribution in % kcal: proteins (17 g/250 mL; 18%), total carbohydrates (47 g/250 mL; 50%), total fats (14 g/250 mL; 32%)]. The MCT is composed of a mixture of 60% octanoic acid (C8) and 40% dodecanoic acid (C10).

### Pharmacokinetic study

Twenty healthy individuals (age 34.7 ± 9.4 years, body weight 64.4 ± 6.7 kg, BMI 21.6 ± 1.8 kg/m^2^, mean ± SD, 13 females and 7 males) were included in this study. Overnight fasted subjects consumed 250 mL of the Peptamen® at time 0 and 4 h. Blood samples (7.5 mL) were taken at regular interval over 8 h [time (min): 0, 15, 30, 45, 60, 120, 180, 240, 255, 270, 285, 300, 360, 420, 480] and plasma was analyzed for total ketone and BHB using Autokit Total Ketone Bodies and Autokit 3-HB (Wako Diagnostics, Mountain View, CA, USA). AcA was then calculated by subtracting BHB to total ketone.

Plasma C8 and C10 free fatty acid levels were measured by LC-MS according to the following procedure: Plasma samples (50 μL) were prepared as previously described (15). Briefly, proteins were precipitated by addition of 450 μL of isopropanol. The suspension was shaken for 30 min and centrifuged for 10 min at 1,500 rpm. 150 μL of supernatant was collected and the solvent evaporated under vacuum. Samples were reconstituted in acetonitrile–water (1:1) and immediately analyzed.

Chromatographic separation was performed on a Waters ACQUITY UPLC BEH C8 Column (1.7 μm, 100 × 2.1 mm) using a linear gradient of water + 0.1% acetic acid (eluent A) and acetonitrile/isopropanol (1:1) + 0.1% acetic acid (eluent B). The gradient was as follow: 0.0–1.0 min at 0% B, 1.0–6.5 min from 0 to 100% B, 6.5–8.5 min 100% B, followed by 2 min of equilibration at initial conditions. Flow rate was set to 450 μL/min, column oven temperature to 55°C and the injection volume to 1 μL. The UPLC system was coupled to an Orbitrap Q Exactive mass spectrometer (ThermoFisher Scientific, Bremen, Germany) operating in negative single ion monitoring mode with a resolving power of 70,000 (at m/z = 200). C8 and C10 free fatty acids chromatograms were extracted using a mass tolerance of 5 ppm and quantified against an external calibration curve in Xcalibur software 2.2 SP1 (ThermoFisher Scientific, Bremen, Germany). Area Under the Curve (AUC) over 0–4 h, and AUC (4–8 h) for ketones, BHB, AcA, C8, and C10 were determined by the trapezoidal method.

### ^31^P-MRS measurement of the human brain

MR experiments were performed on a 7T/68 cm MR scanner (Siemens Medical Solutions, Erlangen, Germany) with an in-house-built ^1^H quadrature surface coil (10 cm-diameter) and a single-loop ^31^P coil (7 cm-diameter) for the coverage of human occipital lobe. Radiofrequency tissue heating parameters were simulated by the finite-difference time-domain method on Sim4Life (ZMT, Zurich Switzerland), and implemented on the scanner. B_0_ field inhomogeneity was optimized in a VOI (50 × 30 × 40 mm^3^) using first- and second-order shimming with FAST(EST)MAP (16).

To measure the forward rate constants for ATP synthesis by creatine kinase and ATP synthase, ^31^P saturation transfer experiment was achieved, using the BISTRO (B1-insensitive selective train to obliterate signal) scheme (17), i.e., a train of hyperbolic secant pulses (50 ms pulse length with a 150 Hz bandwidth) with varied RF amplitude as described herein, precede to a pulse acquire sequence (200 μs hard pulse). The flip angle of the hard pulse was calibrated to achieve maximal signal of PCr prior to the start of acquisition protocols. Four ^31^P acquisitions (spectral bandwidth = 6,000 Hz, 2,048 data points) were performed in the protocol, including (1) a pulse acquire sequence without the saturation transfer scheme (TR = 3 s, average = 320); (2) saturation pulses applied at γ-ATP(−2.5 ppm) with a saturation time τ_sat_ of 8.25 ms (stead-states measurement, TR = 16 s, average = 32); (3) saturation pulses applied at 12.2 ppm (control measurement for correcting the effect of imperfect excitation profile of saturation pulses on Pi, TR = 16 s, average = 32, τ_sat_ = 8.25 ms); (4) saturation pulses applied at 2.5 ppm (control measurement for PCr, TR = 16 s, average = 32, τ_sat_ = 8.25 ms). Representative spectra of saturation transfer experiments were shown in the Supplementary File (Figure 2S). Acquisition 1 is used to measure all ^31^P resonance signals including membrane related metabolites [PE (phophoethanolamine), PC (phosphocholine), GPC (glycerophosphocholine), GPE (glycerophosphoethanolamine), MP (membrane phospholipid)], energy related metabolites [PCr (phosphocreatine), α-ATP, β-ATP, γ-ATP, free phosphate intracellular Pi_int_, and extracellular Pi_ext_], NADH, NAD^+^, intra-/extra-cellular pH and free Mg^2+^ concentrations. Acquisition 2–4 are used to measure forward rate constants of creatine kinase (k_f, CK_) and ATP synthase (k_f, ATPase_).

In summary, the scanning protocol lasted about 60 min including 10 min for preparation procedure (MRI localizer images, shimming and pulse power calibration), 16 min for Acquisition 1 (measurement of NAD, other metabolites and physiological parameters), 9 min for long TR measurement without saturation transfer, and 26 min for Acquisition 2–4 (MT measurement).

### ^31^P MRS quantification

^31^P MR spectra were analyzed by LCModel (18) using a basis-set composed of simulated ^31^P spectra of PCr, α-ATP, β-ATP, γ-ATP, Pi_int_, Pi_ext_, PE, PC, GPC, GPE, MP, NADH, NAD^+^, UPDG (uridine diphosphoglucose) with their respective linewidth (19). [γ-ATP] was assumed to be 3 mM in the human occipital lobe and used as an internal concentration reference. To be comparable with literatures, NAD^+^, NADH, and UDPG concentrations were calculated assuming [α-ATP] of 2.8 mM (12, 13).

Intracellular (pH^int^) and extracellular pH (pH^ext^) values were calculated from the chemical shift difference between Pi^int^/ Pi^ext^ and PCr, respectively (20):

$$\begin{array}{l} pH=pK_{a}+log\frac{\delta_{Pi}-\delta_{a}}{\delta_{b}-\delta_{Pi}} \end{array}$$

where δ_*Pi*_ is chemical shift difference between PCr and Pi; *pKa* = 6.73, δ_*a*_ = 3.275, δ_*b*_ = 5.385.

[Mg^2+^] was calculated from the chemical shift difference between β-ATP and PCr (21):

$$\begin{array}{lll} pMg^{2+} & = & 4.24-\log\left[\frac{{\left(\delta_{\beta ATP}+18.58\right)}^{0.42}}{{\left(-15.74-\delta_{\beta }ATP\right)}^{0.84}}\right]\\ \left[Mg^{2+}\right] & = & {log_{10}}^{{-pMg}^{2+}} \end{array}$$

where δ_β*ATP*_ is the difference in chemical shift between β-ATP and PCr.

Forward rate constants of CK(k_f, CK_) and ATPase(k_f, ATPase_) were calculated from steady-state saturation experiments Mss = Mc/(1+k_f_·T${}_{1}^{\text{int}}$), assuming intrinsic T_1_ of PCr and Pi at 7T are 4.9 s and 3.8 s (22). M_ss_ and M_c_ are signal intensities of Pi or PCr obtained respectively from steady-state and control measurements.

### ^31^P MRS study

Twenty-Five healthy individuals (age 26.6 ± 6.0 years, body weight 68.3. ± 10.4 kg, BMI 22.3 ± 1.7 kg/m^2^, mean ± SD, 8 females and 17 males) were included in this study. Overnight fasted subjects had their first brain ^31^P-MRS scan according to the established protocol above. Pharmacokinetic profile of plasma ketones and free MCFA suggests the maximal values around 30 min and then an additional 15 min was estimated for the ketones to achieve maximum brain concentration and effect. Therefore, they then consumed 250 mL of the Peptamen® over 5 min and waited for a total period of 45 min, after which the second scan was collected. Changes between the 2 scans were calculated and are reported for all parameters in Table 1S (Supplementary Material). The whole experimental protocol is given in Figure 2C.

### Statistical methods

Statistical analysis for the MRS study was done using a mixed model with Peptamen® as fixed effect and subject as random effect. Reproducibility and power calculation to determine the reliability of the measurements by ^31^P MRS was initially investigated in a pilot study in healthy volunteers and is available in the Supplementary Information. We used the data from a preliminary test-retest study with 6 healthy subjects (without product intake) to evaluate intra-subject variation and power calculation. A *t*-test procedure was applied for power calculation, with standard deviation derived from data on 6 healthy subjects and effect size varying between 5 and 20% (in the absence of an intervention product effect in this setting). Based on a range of simulations, we judged 25 subjects to be a sufficient sample for our purposes. A multiplicative effect was assumed and data were log-transformed for analysis. All power calculations were done in R, version 3.3.2, all statistical analyses were done in SAS, version 9.4.

## Results

### Ketones production after MCT intake

**Figure 1:**
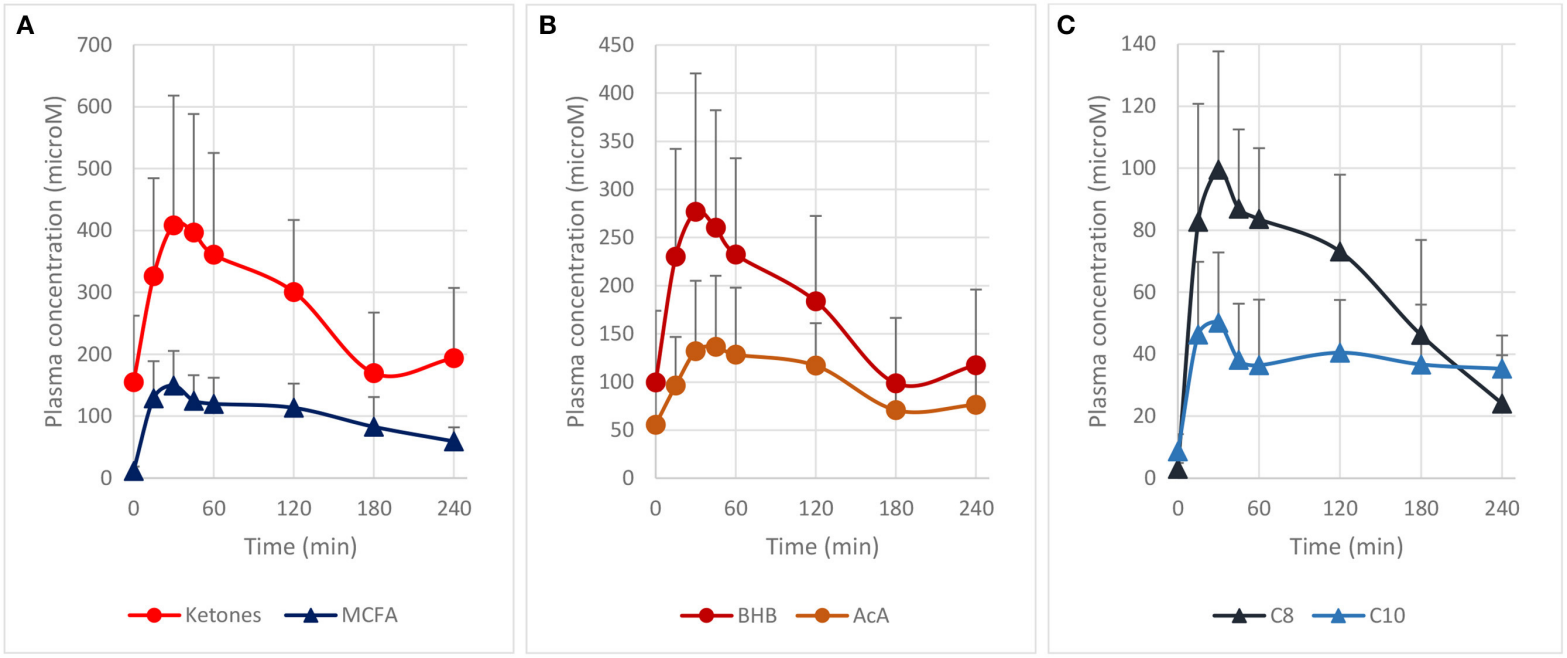
Pharmacokinetic profile of plasma ketones and free MCFA after oral intake of 250 mL Peptamen® containing 10 g of MCT in healthy young volunteers. Values are mean plasma concentration in μM ± standard deviation. Time for maximum concentration (Tmax) around 30 min and a plasma half–life of ~2 h was determined for plasma ketones (BHB and AcA) and free MCFA (C8 and C10). **(A)** Represents total ketones (BHB+AcA) and free MCFA (C8 + C10), **(B)** displays time profile of BHB and AcA, and **(C)** shows free MCFA C8 and C10 plasma concentration (values as mean ± standard deviation).

We first determined the blood ketones pharmacokinetics produced by the ingestion of 10 g of MCT contained in a complete liquid diet. We selected Peptamen® 1.5 Vanilla as an intervention product because it has a high MCT concentration (70% of total fat) in a complete elemental nutrition matrix containing protein, carbohydrate and fat, and mimicking a standard meal rich in MCT. This emulsified formula has demonstrated excellent gastrointestinal tolerability (23), an important consideration as tolerability can be an issue for other MCT preparations (24). After oral intake, high plasma levels of ketones were produced over 2 h (Figure 1A) with a maximum blood ketones concentration (c_max_) of 409 μM. A “keto-index” ratio (corresponding to the total ketones AUC measured over the first 4 h divided by the amount of MCT intake in grams) of 61 μM.h/g was determined and was similar with other MCT formulations tested in analogous conditions (3, 14, 24). Both BHB and AcA increased proportionally (Figure 1B), with a BHB/AcA ratio of 1.8 ± 0.3 across the 4 h. The free MCFA C8 and C10 plasma concentrations, precursors of the ketones, were also determined, since they can cross the blood-brain-barrier through passive diffusion (25–27) and be metabolized to ketones as well (28, 29). MCFAs displayed a similar kinetic profile to the ketones supporting concomitant metabolism of the free MCFA by the liver, with a maximum concentration of 150 μM at 30 min (Figure 1C). The free MCFA C8 had a greater plasma concentration (AUC_0−4h_ 219 μM.h) than C10 (AUC_0−4h_ 116 μM.h), consistent with the larger proportion of C8 (60%) in the initial MCT composition. It is unclear why the MCFA C10 did not return fully to baseline after 4 h. The increase in ketones and MCFA were highly reproducible as a second oral intake of Peptamen® after 4 h lead to similar results (data not shown), with only minimal plasma accumulation. Overall, this study demonstrated that significant nutritional ketosis can be established over 2 h by the oral intake of Peptamen® containing 10 g of MCT, with a maximum plasma effect at around 30 min.

### Brain metabolites changes measured by ^31^P MRS

^31^P MRS at high magnetic field is a powerful non-invasive method to measure metabolic and physiological parameters in the brain (30) including important ^31^P-metabolites involved in brain energy metabolism (PCr, Pi, ATP) and membrane synthesis/degradation (PC, PE, GPC, GPE), metabolic fluxes (k_f, CK_ and k_f, ATPase_), intra-/extra-cellular pH, free Mg^2+^ concentration, as well as most recently the redox state (NAD^+^/NADH) (11–13). To measure the aforementioned metabolic and physiological parameters with a focus on redox state and forward rate constants of CK(k_f, CK_) and ATPase(k_f, ATPase_), a scanning protocol of the occipital region was established in a pilot test-retest study in 6 healthy volunteers (method section and Supplementary Material). Measurement of the NAD^+^ and NADH by ^31^P MRS is especially challenging due to their intrinsically low concentration and strong spectral overlap with each other. Therefore, the current ^31^P MRS measurements were performed at 7T where NAD content has already been successfully reported using ^31^P MRS (11–13) with enhanced sensitivity and spectral resolution. The quantification of NAD^+^ and NADH was performed using LCModel as a quantification method. The results of Monte-Carlo simulations suggest high accuracy of the measurement (Supplementary Material). The power calculation derived from the pilot study indicated that with 25 subjects used in the current study, the method is sensitive for the detection of less than 5% changes for NAD^+^, and about a 10% change for NADH and redox ratio NAD^+^/NADH.

**Figure 2:**
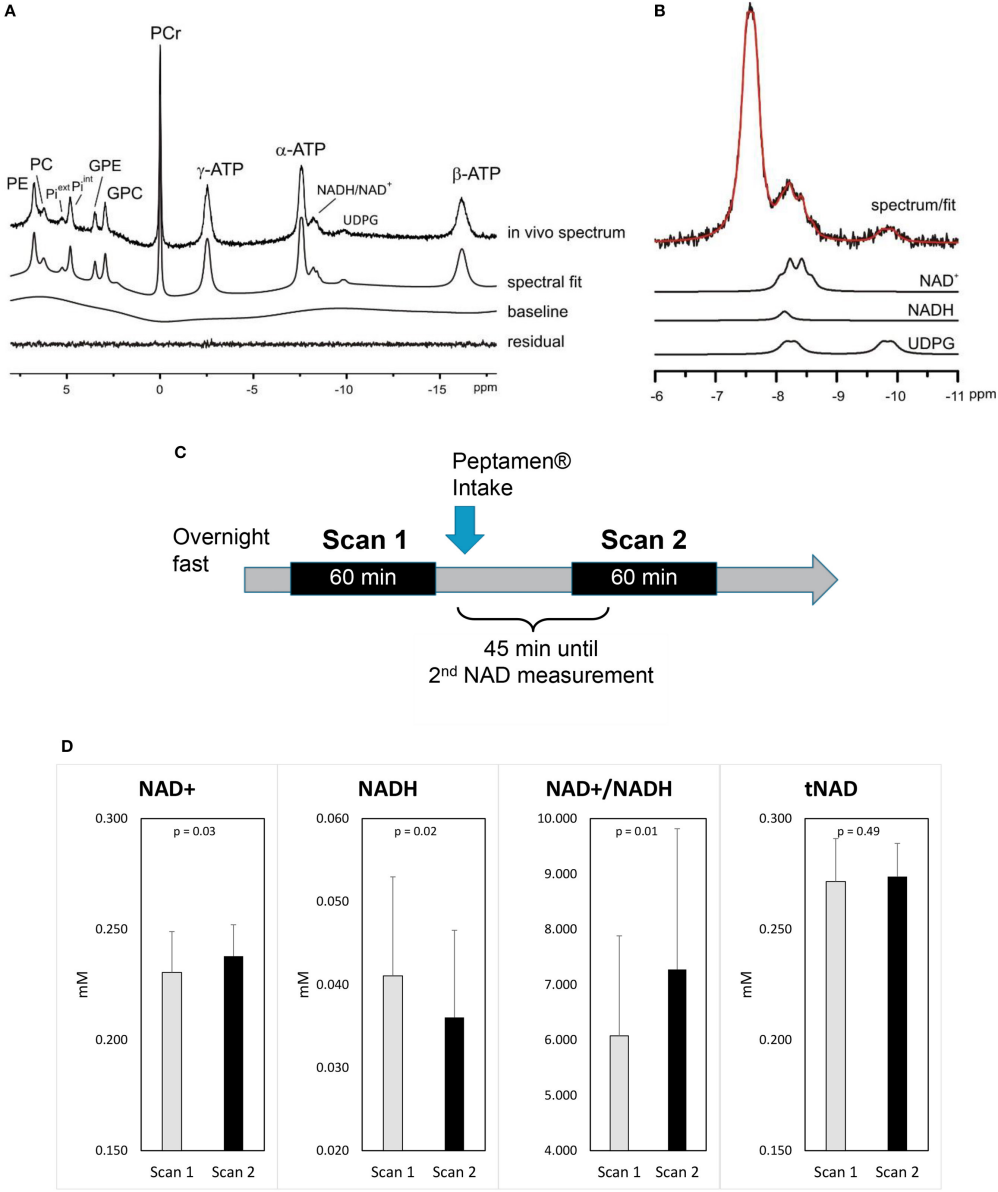
Impact of ketones on brain metabolism **(A)** a representative ^31^P MRS spectrum acquired from the occipital lobe using a surface coil (no baseline correction and no apodization were applied). LCModel fit of the *in vivo* spectrum **(A)** and **(B)** individual fits of NAD^+^, NADH, UDPG are shown. Excellent fitting quality is suggested by the minimum residual. **(C)** Study design. **(D)** Changes in brain NAD concentrations (values as mean ±standard deviation); *p*-values shown are derived from the mixed model.

A typical ^31^P MRS spectrum acquired in the occipital of a healthy volunteer is shown in Figures 2A,B including its spectral fit obtained from LCModel and individual fits of NAD^+^, NADH, and UDPG.

After the intake of Peptamen®, the main observation was a significant change in NAD metabolites levels: NAD^+^ was increased by 3.4% while the level of NADH was reduced by 13% resulting in a 18% increase of the redox ratio NAD^+^/NADH (*p* = 0.01; Figure 2D). To further demonstrate that the variations in NAD levels were independent of the fitting model, differential averaged spectra (scan2-scan1) for several volunteers resulted in a non-flat difference spectra in the NAD region (see Supplementary Material), supporting an elevated signal for NAD^+^ and a diminished signal for NADH after the intake of Peptamen®. No change in PCr or Pi levels or their ratios could be detected (see Supplementary Material), nor changes in the metabolic fluxes for ATP production from PCr or Pi.

## Discussion

**Figure 3:**
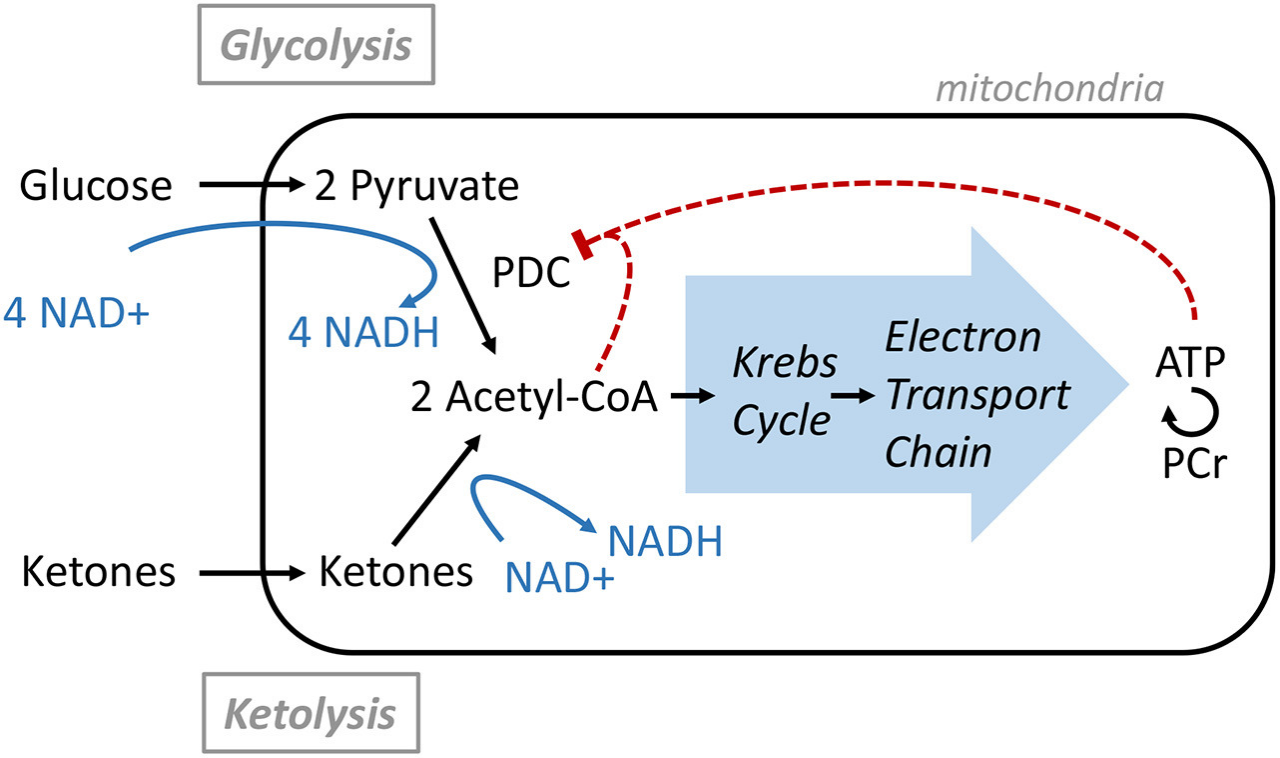
Acetyl-CoA production from Glucose or Ketone and its regulation.

Overall, this study demonstrated that significant nutritional ketosis can be maintained for 2 h by oral Peptamen® intake with a maximum plasma concentration at about 30 min. Under these conditions, the brain NAD^+^/NADH ratio increases together with increasing level of NAD^+^ and decreasing level of NADH. These are the only brain metabolism parameters that were significantly affected as measured by ^31^P-MRS. This finding confirms the results obtained in brain of rodents where an increase in NAD^+^/NADH ratio was observed after ketones exposure (8, 9). The NAD^+^ requirement of the glycolytic and ketolytic pathways is illustrated in Figure 3. Glycolysis requires 4 NAD^+^ to make 2 Acetyl-CoA, while ketolysis consumes no NAD^+^ to make Acetyl-CoA from AcA, and only one NAD^+^ to convert BHB to AcA (6). This is to the best of our knowledge the first interventional study that shows that ketones can have a NAD^+^ sparing effect in healthy human brain.

That no other metabolic change could be detected supports previous brain studies in healthy humans demonstrating that ketones can replace a portion of the glucose utilized by the brain while maintaining brain energy homeostasis (4). Ketones are actively taken up by the brain via the MCT1 and their levels are directly correlated to their plasma level with no apparent saturation (2, 3). In healthy resting conditions, the energy equilibrium is maintained by a feedback loop mechanism at the level of the pyruvate dehydrogenase complex (PDC; Figure 3); the downstream metabolites such as ATP and Acetyl-CoA act as negative regulators (6). As more ketones are metabolized in the mitochondria to form Acetyl-CoA, less glucose is utilized.

A decline in NAD^+^ availability and abnormal NAD^+^/NADH redox state has been directly linked to age-related metabolic diseases and neurodegenerative disorders (10). However, their direct measurements by ^31^P-MRS in human brain have only recently been made possible by use of high magnetic fields (11–13). A 25–55% decrease in the NAD^+^/NADH ratio has been reported in elderly (frontal and occipital lobes) and in psychiatric disorders such as schizophrenia and bipolar disorder (frontal lobes), together with a reduction in total NAD. Using a similar approach, our study shows that ketones can increase the brain NAD^+^/NADH ratio by about 18% in humans, at least transiently. Therefore, a nutritional ketone intervention, beyond providing an alternative source of energy to the brain, offers the potential to boost the NAD^+^/NADH redox state and may provide additional benefits to the brain such as protection from oxidative stress and inflammation (2, 7).

The pharmacokinetic profile of the MCT contained in Peptamen® is consistent with the published human data indicating that MCT is rapidly digested and primarily metabolized by the liver to form significant levels of blood BHB and AcA (3, 24). MCT-C8 has been shown to be the main driver of ketone formation, while MCT-C10 does not produce substantial ketosis (14). Interestingly, we observed a significant level of free MCFA C8 and C10. Free C8 and C10 have been shown to cross the blood-brain barrier in rodent by passive diffusion reaching a brain level ~1.5 to 10 fold lower concentration than in blood (25–27) where both seems to be extensively metabolized (28, 29). Therefore, we should not rule out that free MCFA C8 and C10 could contribute directly to some brain energy metabolism. These data, combined with the observation that C10 might be less prone to brain beta-oxidation than C8 (31) suggest that further experiments with MCT-C10 in human might reveal unsuspected metabolic effects (32).

The lack of a control group without MCT is the main limitation of the current study, and we cannot rule out a contribution of the food matrix to the results, although an increase in brain glycolysis driven by the intake of carbohydrates should lead to a transient decrease in NAD^+^ (33). We demonstrated a significant increase of NAD^+^/NADH ratio in the brain following acute blood ketones generation, yet the effect of chronic ketones exposure remains to be explored in a placebo controlled study. Moreover, it is currently not possible to differentiate NAD metabolism between cell types, nor cytosolic vs. mitochondrial using ^31^P MRS. While the data generated here are in healthy brain, it will also be important to investigate if ketones have similar effects in cohort with NAD^+^/NADH deficits such as elderly subjects, and how this effect may be linked to clinical outcomes. Remarkably, Glucose transporter type 1 deficiency syndrome patients treated with MCT experienced improvement of their symptoms and a 10% increase of their brain Pi/PCr ratio during visual stimulation as measured by ^31^P-MRS (34). This suggests that relatively small changes in metabolite concentrations can translate into significant clinical benefits, further supporting the use of ketones for the treatment of brain energy deficit (2, 4, 7). The use of other ketones precursors such as ketone esters or ketone salts (35) might provide greater acute blood ketones concentrations and should also be investigated.

Note that the measurement of NAD^+^ and NADH by ^31^P MRS is challenging due to their limited spectral separation. Specifically, at 7T the NADH resonance is overlapping with the downfield part of the NAD^+^ quartet. Thus, the separation of NAD^+^ and NADH relies on the right half side of NAD^+^ quartet, which is discernible in the spectral region of NAD in the current study (Figure 2C, Figure 3S) and another 7T study (11, 36). This implies that excellent shimming performance is important to allow the separate measurement of NAD^+^ and NADH. On the other hand, measurement of NAD^+^ has been recently reported using 1H MRS (36), which may serve as an alternative for further validation of the result by 31P MRS.

## Conclusion

In conclusion, the present study suggests that ketones might indirectly exert a neuroprotective role via an elevation of the NAD^+^/NADH ratio. In healthy humans chronic supplementation of ketone precursors such as MCT may reduce brain glucose utilization, and could result in a protective effect via preservation of the various NAD metabolic pathways involved in health and repair. Although such a nutritional ketosis approach will require further investigations, it might be as efficacious as ketogenic diets, caloric restriction regimens, or intermittent fasting in preserving brain health.

## Study approval

Both studies were approved by the Ethics Committee of Canton de Vaud (Switzerland) under the references 341/14 and 2017-00159, respectively, and all participants provided written informed consent. Procedures were conducted according to the principles of the Declaration of Helsinki. Trial registration numbers: NCT02241681 and NCT03101345, respectively.

## Author contributions

LX, ÖI, RG, MB, MS, NG, and BC designed the studies. Data collection was performed by LX, ÖI, NC, MM, and MB. MS analyzed data. The manuscript was drafted by BC and all authors discussed the results and revised the manuscript.

### Conflict of interest statement

MB, MS, NG, NC, MM, MS, and BC are employees of Nestlé. The remaining authors declare that the research was conducted in the absence of any commercial or financial relationships that could be construed as a potential conflict of interest.
