# Supplementary material for: Nutritional Ketosis Increases NAD+/NADH Ratio in Healthy Human Brain: An in Vivo Study by 31P-MRS
Source: Front Nutr. 2018 Jul 12;5:62. doi: 10.3389/fnut.2018.00062 (PMC6052097; doi:10.3389/fnut.2018.00062)
Supplement: Supplementary file 1 [file Presentation_1.PDF]

Supplementary Information

Nutritional ketosis increases NAD+/NADH ratio in healthy human brain: an *in vivo* study by ^{31}P-MRS

Authors

Lijing Xin*, Özlem Ipek, Maurice Beaumont, Maya Shevlyakova, Nicolas Christinat, Mojgan Masoodi, Norman Greenberg, Rolf Gruetter, Bernard Cuenoud*

Accuracy of NAD+ and NADH measurement: Monte Carlo simulations

Monte Carlo simulations were performed to evaluate the impact of spectral quality (i.e., SNR and linewidth (LW)) on the measurement accuracy. Simulated ^{31}P spectra were generated with different SNR_{PCr} (30-240, 30/step) and LW_{PCr} (6.5-12.5 Hz, 1Hz/step) at 7T using the published chemical shift, J-coupling constants and concentrations (1) . A baseline obtained by a spline fit of summed in vivo spectra was added into the simulated spectra. One hundred spectra were generated at different experimental conditions by adding random Gaussian noise. Measurement accuracy was assessed by comparing the quantified values with its respective true values using the estimation error(%)=([meas]-[true])×100/[true].

Results of Monte Carlo simulations (Figure 1s) demonstrated excellent accuracy of measurement when $SNR_{PCr}>50$ and the measurement precision (sd of the estimation error) is improved with the increase of SNR and converged beyond SNR of 100. In addition, the spectral linewidth shows very minor impact on both the accuracy and precision. The SNR_{PCr} (166±29 for scan1, 170±33 for scan2, p=0.86 paired two-tailed t-test) and LW_{PCr} (9.5±1.1Hz for scan1, 9.5±1.1Hz for scan2, p=0.30) of both scan1 and scan2 demonstrated comparable spectral quality, which allows the measurement of NAD^+, NADH and redox ratio with the accuracy of -0.9 ± 3.3%, 2.6±9.9% and -2.4±11.7%, respectively.

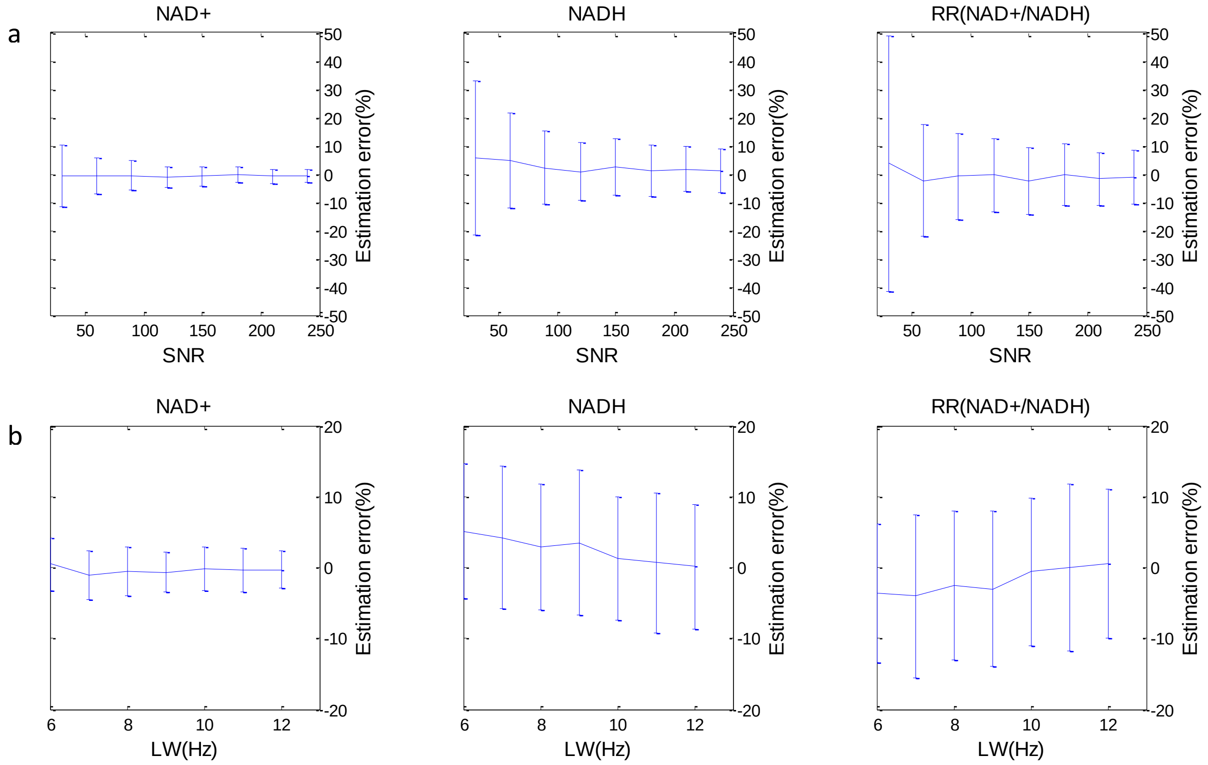

Figure 1s. The effect of SNR_{PCr}(a) and LW_{PCr}(b) on estimation errors of NAD+, NADH and RR using Monte Carlo simulations. Simulated spectra were generated with a) SNR_{PCr} = 30-240 and LW_{PCr} of 9.5Hz; b) LW_{PCr}=6.5-12.5Hz and SNR_{PCr} of 160. Estimation errors are shown as mean values with error bars indicating the standard deviations of the estimation errors.

Detailed information about the magnetization transfer experiment for metabolic rate measurement.

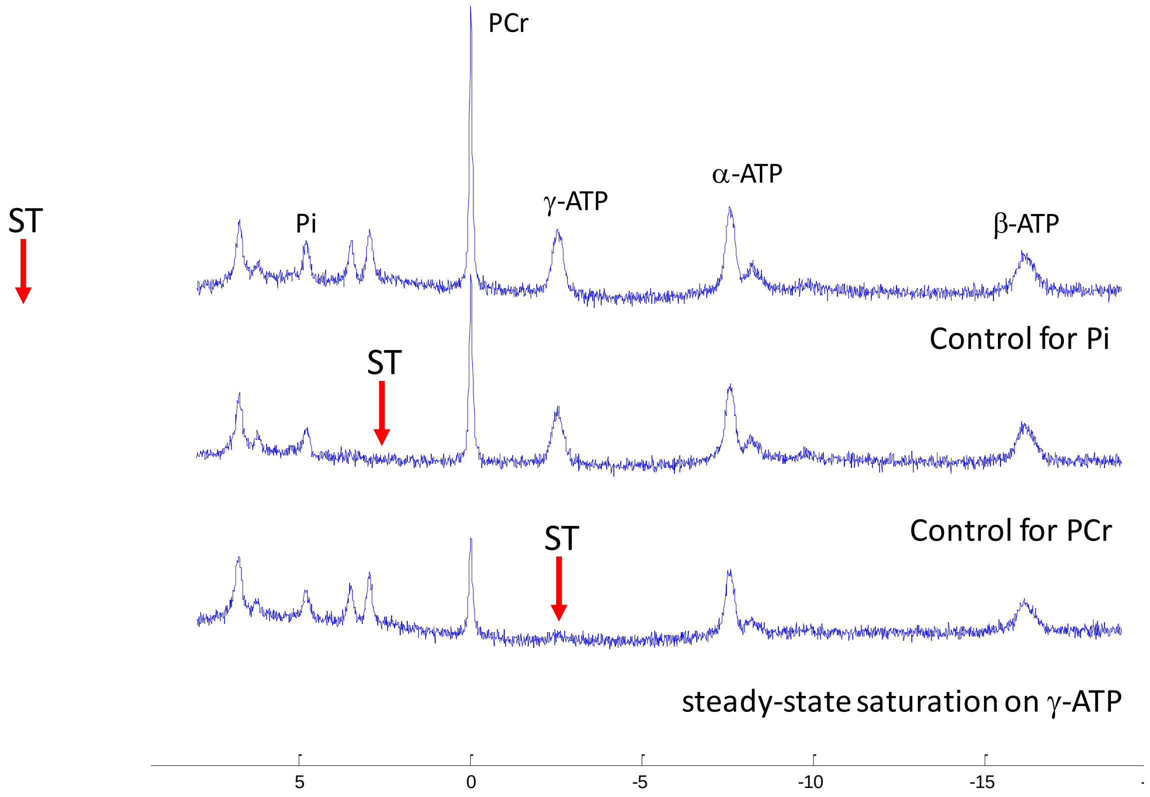

Figure 2s. ^{31}P MR spectra acquired with saturation pulses (τ_{sat} =8.25ms) applied at 12.2ppm (control measurement for Pi, top), at 2.5ppm(control measurement for PCr) and at γ-ATP resonance (-2.5ppm, bottom).

Reproducibility and the power calculation

Six healthy volunteers (4M/2F, 19-33y) gave informed consent prior to the study and were recruited for a test-retest study to evaluate the reproducibility and the power of the measurement of the various metabolites and rate constant, with an emphasize on NAD content. The same scans as described in the methods part were applied two times for these subjects without product uptake. The main measurement results were listed in the table 1s and show very low intra-subject variability. Based on these data we performed a range of simulations for sample size assessment and power calculations (done via a t-test procedure with standard deviation estimated from a mixed model on our data). For effect sizes ranging between 5% and 20% corresponding samples sizes were estimated for a range of parameters (see table 2s). We can see that for NADH and for the redox ratio RR (NAD^+/NADH) measurements we can expect to detect a 10% change within 25 subjects. And for NAD^+, 25 subjects will allow the power to detect less than 5% changes. In our actual experiment the effect that we detect on NAD+, NADH and RR are 3.4%, 13% and 18%, respectively, which are in line with power estimations prior to the study, especially since we use a mixed model for analysis which is slightly more powerful than a t-test used for sample size calculations.

	Concentrations (mM)		Scan2-Scan1[mM]	p-value
	Scan 1	Scan 2		
**NAD^+**	0.233 ± 0.005	0.235 ± 0.013	0.002 ± 0.009	0.64
**NADH**	0.043 ± 0.007	0.044 ± 0.004	0.0015 ± 0.0061	0.50
**NAD^+/NADH [-]**	5.6 ± 1.0	5.3 ± 0.5	-0.27 ± 0.70	0.51
**UDPG**	0.180 ± 0.010	0.166 ± 0.020	-0.014 ± 0.017	0.10
**PCr**	2.805 ± 0.177	2.809 ± 0.102	0.004 ± 0.115	0.88

Table 1s. Concentration, difference (scan2-scan1) and p-value of NAD^+, NADH, redox ratio RR(NAD+/NADH) and UDPG measured by LCModel from *in vivo* ^{31}P MR spectra in human occipital lobe at 7T. Results are reported in mean ± std (n=6).

Effect size	5%		10%		20%	
power	80%	90%	80%	90%	80%	90%
NADH	63	84	18	24	7	8
NAD+	8	10	4	5	3	3
RX	63	83	18	24	7	8
UDPG	35	46	11	14	5	6
PCr	11	13	5	6	3	4

Table 2s: sample size estimation for NAD+, NADH, RR and UDPG assuming 5%, 10% and 20% effect size

Differential averaged spectra (scan2-scan1)

To further demonstrate that the variation in NAD levels and redox ratio are independent of the fitting model, we calculated the differential averaged spectra (scan2-scan1) for six volunteers with large changes in NAD. The summed spectra obtained before and after Peptamen intake were overlaid after normalization of peak intensity to α-ATP (internal reference) and spectral frequency alignment. Increased NAD+ after Peptamen intake leads to higher signal on the right side shoulder of the NAD+ quartet spectra, while the left side resonance peaks were compensated by the reduction of NADH, showing small decrease in apparent spectral difference. The fact that NAD+ increase and NADH decrease simultaneously, with no change in total NAD concentration limits the differential spectra approach reported in (2).

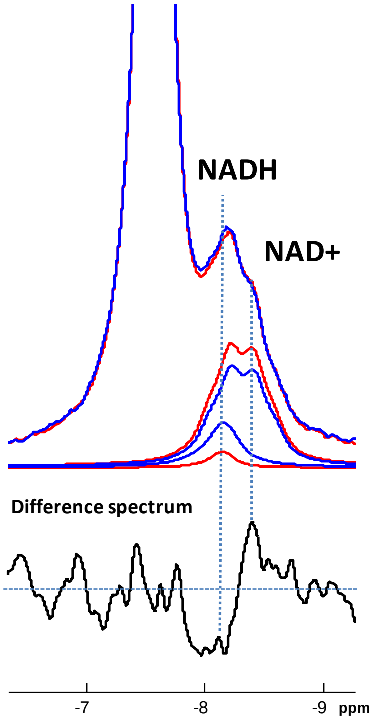

	before	after
NAD+	0.223 ± 0.022 mM	0.239 ± 0.021 mM
NADH	0.048 ± 0.016 mM	0.028 ± 0.007 mM
RR	5.07 ± 1.64	9.01 ± 2.65

Figure 3s. Summed spectra of 6 subjects (with >50% change in NAD+/NADH ratio) acquired before (blue) and after (red) Peptamen intake are overlaid. LCModel fit for NAD+ and NADH are also displayed. The difference spectrum of before and after Peptamen intake is shown at the bottom.

Table 3s. ^{31}P metabolites concentrations and calculated parameters obtained before (scan1) and after (scan2) uptake of the product.

	scan1		scan2		
**Concentrations[mM]**	**mean**	**sd**	**mean**	**sd**	**P value**
**NADH**	0.041	0.012	0.036	0.011	0.02
**NAD^+**	0.231	0.018	0.238	0.014	0.03
**UDPG**	0.163	0.018	0.158	0.013	0.26
**tNAD**	0.272	0.019	0.274	0.015	0.49
**RR(NAD^+/NADH) [-]**	6.1	1.8	7.3	2.5	0.01
**PCr**	2.99	0.17	3.03	0.14	0.18
**Pi_int**	0.89	0.07	0.90	0.06	0.52
**PC**	0.62	0.08	0.64	0.08	0.28
**PE**	1.33	0.07	1.36	0.07	0.03
**GPC**	0.92	0.09	0.91	0.08	0.45
**GPE**	0.59	0.05	0.58	0.04	0.33
**MP**	0.33	0.08	0.31	0.07	0.10
**Pi_ext**	0.12	0.06	0.12	0.06	0.50
**PME(PE+PC)**	1.96	0.11	2.00	0.11	0.04
**PDE(GPC+GPE)**	1.51	0.13	1.49	0.12	0.35
**Caculated parameters**					
**pH_int [-]**	6.99	0.01	6.99	0.00	0.51
**pH_ext[-]**	7.39	0.01	7.39	0.01	0.94
**[Mg^{2+}] (mM)**	0.168	0.004	0.168	0.004	0.97
**Mss/Mc(PCr) [%]**	47	2	48	3	0.28
**Mss/Mc(Pi) (%)**	67	5	70	5	0.12
**k(PCr->ATP) [s^{-1}]**	0.230	0.022	0.224	0.028	0.27
**k(Pi->ATP) [s^{-1}]**	0.131	0.029	0.118	0.028	0.14

References

1. Lu M, Zhu XH, Zhang Y, Chen W. Intracellular redox state revealed by in vivo (31) P MRS measurement of NAD(+) and NADH contents in brains. *Magn Reson Med* 2014; 71(6)**:** 1959-72.
2. Kim SY, Cohen BM, Chen X, Lukas SE, Shinn AK, Yuksel AC *et al.* Redox Dysregulation in Schizophrenia Revealed by in vivo NAD+/NADH Measurement. *Schizophrenia bulletin* 2017; 43(1)**:** 197-204
